# Comparative Transcriptome Profiling of Two Tomato Genotypes in Response to Potassium-Deficiency Stress

**DOI:** 10.3390/ijms19082402

**Published:** 2018-08-14

**Authors:** Xiaoming Zhao, Yang Liu, Xin Liu, Jing Jiang

**Affiliations:** 1 The Key Laboratory of Protected Horticulture Ministry of Education, College of Horticulture, Shenyang Agricultural University, Shenyang 110866, China; 04012020zxm@163.com (X.Z.); yang_liu@celestialseeds.com (Y.L.); 2 College of Agriculture, Jilin Agriculture Science and Technology College, Jilin 132101, China

**Keywords:** tomato, genotypes, transcriptome, potassium deficiency stress, DEGs, root configuration

## Abstract

Tomato is a crop that requires a sufficient supply of potassium (K) for optimal productivity and quality. K^+^-deficiency stress decreases tomato yield and quality. To further delve into the mechanism of the response to K^+^-deficiency and to screen out low-K^+^ tolerant genes in tomatoes, BGISEQ-500-based RNA sequencing was performed using two tomato genotypes (low-K^+^ tolerant *JZ34* and low-K^+^ sensitive *JZ18*). We identified 1936 differentially expressed genes (DEGs) in *JZ18* and *JZ34* at 12 and 24 h after K^+^-deficiency treatment. According to the Gene Ontology (GO) and Kyoto Encyclopaedia of Genes and Genomes (KEGG) pathway analyses, the DEGs that changed significantly primarily included transcription factors, transporters, kinases, oxidative stress proteins, and hormone signaling-and glycometabolism-related genes. The experimental results confirmed the induced expression of the responsive genes in the low-K^+^ signaling pathway. The largest group of DEGs comprised up to 110 oxidative stress-related genes. In total, 19 ethylene response factors (ERFs) demonstrated differential expression between *JZ18* and *JZ34* in response to K^+^-deficiency. Furthermore, we confirmed 20 DEGs closely related to K^+^-deficiency stress by quantitative RT-PCR (qRT-PCR), some of which affected the root configuration, these DEGs could be further studied for use as molecular targets to explore novel approaches, and to acquire more effective K acquisition efficiencies for tomatoes. A hypothesis involving possible cross-talk between phytohormone signaling cues and reactive oxygen species (ROS) leading to root growth in *JZ34* is proposed. The results provide a comprehensive foundation for the molecular mechanisms involved in the response of tomatoes to low K^+^ stress.

## 1. Introduction

K^+^ is required in most plants as a cationic mineral nutrient [1,2]. This element is essential for many physiological processes, including photosynthesis, enzyme activation, protein synthesis, osmoregulation, cell turgor, and ion homeostasis in plant cells [3]. Plant growth requires K^+^, but most plants can absorb only a minimal amount of soluble K^+^ from the soil [4]. K^+^-deficiency can directly disturb physiological activities and restrict assimilation and partitioning into fruits from the nutrient source, resulting in decreased crop growth, and fruit production and yield [5]. Increasing the utilization of the K^+^ nutrient or the resistance of the crop itself to K^+^ deficiency is the major method that can solve the K^+^-deficiency problem. However, the molecular mechanisms responsible for these differences are not clear.

K^+^ is primarily absorbed by plant roots and is transported to the root surface from the soil [6]. Root characteristics are the most important factors determining K^+^ uptake; for instance, in cotton, the tap root length and lateral root (LR) numbers were significantly higher in high-efficiency genotypes than in low-efficiency genotypes [7]. Rice crops maintain K^+^ uptake by modifying their root hair length under K^+^-deficient conditions [8]. An excellent root system is the basis for absorbing more nutrients, but whether the lack of K^+^ in tomatoes induces the expression of related genes and thus promotes root structure changes to adapt to K^+^ deficiency, is unclear. Changes in root architecture represent an adaptation of the external shape of the plant in response to nutrient deficiency. In addition, plants in poor nutrient conditions will alter the expression of many related genes to adapt to the adverse environment. K^+^ transporters (high affinity) and K^+^ channels (low affinity) transport K^+^ from the soil to plant cells [9]. In the tomato, 19 K^+^ channels and transporters have been identified [10]. High-affinity K^+^ transporters play very important roles under K^+^-deficient conditions. Different genotypes express various high-affinity K^+^ transporters with diverse K^+^ uptake efficiencies [11]. K^+^-deficiency stress can induce the production and alteration of many recognized signal substances, such as transcription factors, reactive oxygen species (ROS), plant hormones, kinases, and carbohydrates [12,13], and differences in the expression of these genes in different genotypes have great significance.

We previously identified the tomato cultivar *JZ34* as a K^+^-efficient genotype, whereas cultivar *JZ18* was sensitive to low K^+^, and was considered a K^+^-inefficient genotype. A more highly developed root system, and better K^+^ uptake ability were observed under K^+^-deficiency in *JZ34* than in *JZ18* [14]. Understanding how tomato plants regulate K^+^ transport systems via gene networks, to acclimate to K^+^-deficiency is valuable. Although some transcriptome studies of responses to K^+^-deficiency have been published in Arabidopsis, rice, tobacco, and other plants [15,16,17,18,19], no information on genome-wide transcription levels in tomatoes is available. Comparative transcriptome analysis of K^+^-efficient and K^+^-inefficient genotypes can more accurately identify key genes responsive to K^+^ deficiency. In the present study, an RNA-seq approach was applied to hydroponically grown *JZ34* and *JZ18* tomatoes after K^+^ deprivation for 12 and 24 h to explore differences in the early responses in the transcriptome profiles of K^+^-efficient and K^+^-inefficient genotypes. Temporal patterning expression and the regulation of genes by K^+^ deprivation was comprehensively characterized. This study is the first to present a global transcriptome analysis of two tomato genotypes, and to provide new insights into the molecular mechanisms underlying the tolerance of the K^+^-efficient genotype to K^+^-deficiency.

## 2. Results

### 2.1. Analysis of Root Systems in JZ34 and JZ18 under K^+^-Deficient Conditions

**Figure 1:**
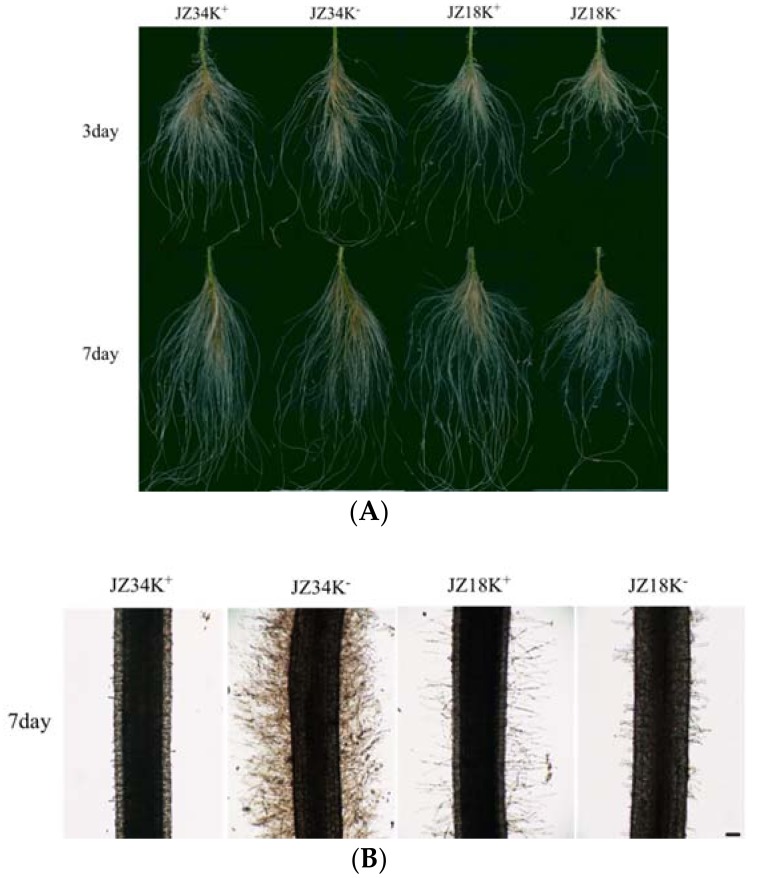
Comparison of morphological changes in the roots of different tomato genotypes under K^+^-deficiency stress conditions at 3 d and 7 d (**A**); changes in the root hair region in different tomato genotypes under K^+^-deficiency stress conditions at 7 d. Roots were observed at 100 × magnification; scale bars: 0.01 cm (**B**). K^+^ represents normal K^+^ (4 mM); K^−^ represents K^+^ deficiency (0 mM). The experiments were repeated three times.

Two tomato genotypes (low-K^+^ tolerant *JZ34* and low-K^+^ sensitive *JZ18*) were used to investigate different genotype responses to K^+^-deficiency that exhibited marked differences in sensitivity to K^+^-deficiency and root morphology. In *JZ18*, K^+^-deficiency stress reduced the total number of LRs and root hairs. In contrast, the numbers of LRs in *JZ34* were similar under both low and normal K^+^ concentrations, and more root hairs were found at the low K^+^ concentration (0 mM) than at the normal K^+^ concentration (4 mM) (Figure 1). The morphological responses implied that *JZ34* had a stronger nutritional uptake capability than did *JZ18*. The biomasses of these two tomato varieties under the K^+^-deficient (0.5 mM) condition changed differently in this experiment (Table 1). The relative coefficients of the dry and fresh weight were 1.03 and 1.07 in *JZ34*, and 0.96 and 0.71 in *JZ18*, respectively.

**Figure 2:**
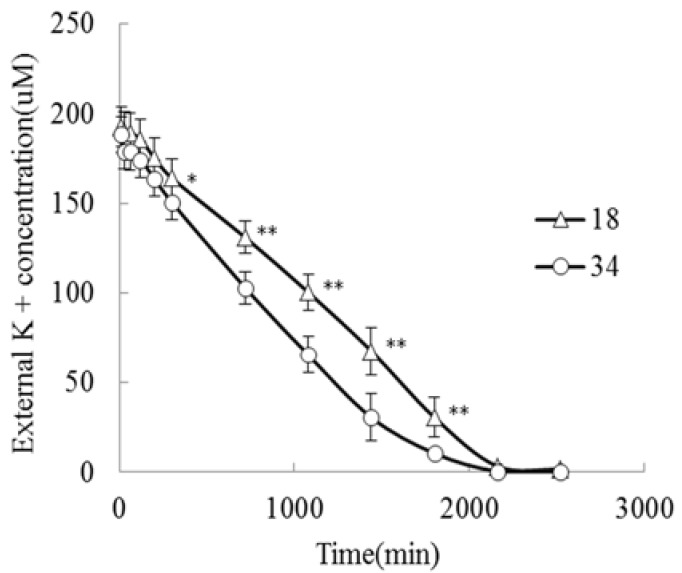
The K^+^ uptake ability was compared using K^+^ depletion between two different genotypes. The experiments were repeated three times. * and ** denote significant differences at *p* < 0.05 and *p* < 0.01, respectively.

K^+^ can increase crop yields and improve crop quality. The K^+^ concentrations in the K^+^-deprived seedlings of both genotypes declined after 7 d under K^+^-deficiency treatment. Nevertheless, *JZ34* maintained a higher K^+^ content under K^+^-deficiency stress, indicating that this cultivar was better at maintaining a high K^+^ concentration under K^+^-deficiency than was *JZ18*. The K^+^ content of *JZ34* declined slightly under these conditions, with a relative coefficient of 0.89. However, the K^+^ content in *JZ18* dropped significantly, with a relative coefficient of 0.57. K^+^ accumulation still trended similarly. To determine the K^+^ uptake ability of both genotypes, K^+^ was depleted in the uptake solution, which reflected its net uptake by the roots monitored over 30 h. External K^+^ was depleted to a greater extent by *JZ34* than by *JZ18* (Figure 2). We determined the maximum ion absorption rate (I_max_) and Michaelis-Menten constant (K_m_) in the presence of 0.2 mM external K^+^. These two parameters quantitatively described the ability of the plants to absorb nutrient ions (*JZ34*: I_max_ = 0.10 mmol·g^−1^·min^−1^; K_m_ = 0.107 mmol/L; *JZ18*: I_max_ = 0.06 mmol·g^−1^·min^−1^; K_m_ = 0.125 mmol/L). The K^+^-uptake ability of *JZ34* was better than that of *JZ18* [20].

### 2.2. Overview of the Gene Expression Profile Sequence Data and Mapping Results

To obtain a global overview of the transcriptome relevant to different K^+^-deficiency stress conditions in the tomato, we separately sequenced complementary DNA (cDNA) libraries from root samples of *JZ34* and *JZ18* at different time points under K^+^-deficiency (0.5 mM) using the BGISEQ-500 platform. After the removal of low-quality reads, an average of 2.4 × 10^7^ clean reads was obtained for each sample, and the total length of the clean reads reached 1.2 × 10^9^ nt. For each sample, 99% of the clean reads were mapped to the tomato reference transcriptome (Table 2).

The expression levels of the mapped genes were normalized using FPKM values. To confirm the quality of the RNA-seq results, we evaluated the expression of the 12 highest-ranking housekeeping control genes in tomatoes at both 12 and 24 h, including GAPDH, catalase, Cys protease, α-tubulin, ubiquitin, actin, DNAJ, and translation initiation factor 5A (Table 3). None of these reference genes were significantly differentially expressed in the *JZ34* and *JZ18* lines by pairwise comparison after the induction of K^+^ deficiency (Table 3), indicating that the obtained sequences and transcript levels were suitable for further transcriptome analysis.

**Figure 3:**
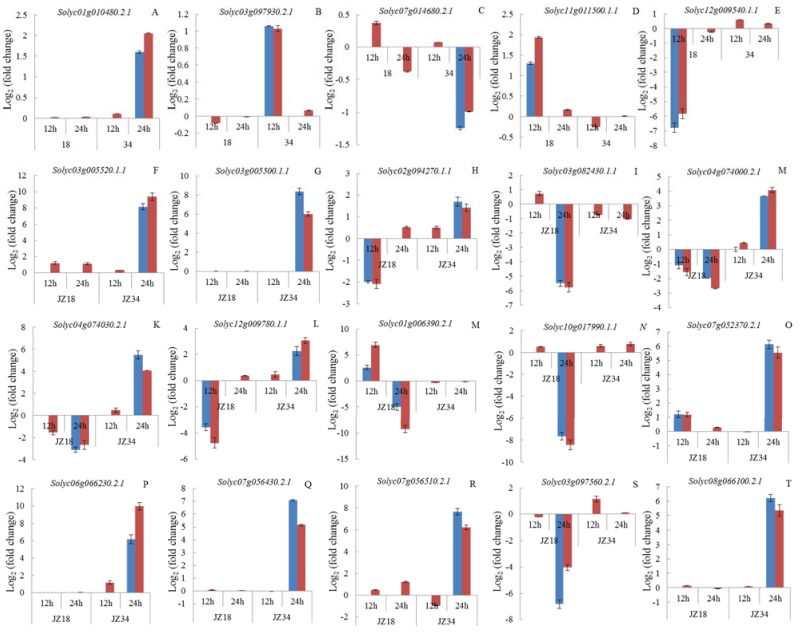
Quantitative real-time PCR validation of 20 differentially expressed genes (DEGs). The blue column represents RNA-seq data, and the red column represents real-time PCR data. The experiments were repeated three times. The genes (**A**–**T**) function annotations are shown Table S1.Gene-specific primers used for real-time PCR are listed in Table S5.

To clarify the transcriptome results, we chose 20 genes for quantitative RT-PCR (qRT-PCR) analysis (Figure 3). The genes included those associated with K^+^ channels (*Solyc01g010480.2.1*, *Solyc03g097930.2.1*, *Solyc07g014680.2.1*, *Solyc11g011500.1.1*, and *Solyc12g009540.1.1*) (Figure 3A–E), transcription factors (*Solyc03g005520.1.1*, *Solyc03g005500.1.1*, *Solyc02g094270.1.1*, and *Solyc03g082430.1.1)*, kinases (*Solyc04g074000.2.1*, *Solyc04g074030.2.1*, *Solyc12g009780.1.1*, and *Solyc01g006390.2.1*), hormones (*Solyc10g017990.1.1*), oxidative stress (*Solyc07g052370.2.1, Solyc06g066230.2.1*, *Solyc07g056430.2.1*, and *Solyc07g056510.2.1*), glycometabolism (*Solyc03g097560.2.1* and *Solyc08g066100.2.1*) (Figure 3F–T). The gene function annotations are shown Table S1. The selection criteria for DEGs (F–T) were a difference in the expression levels between two genotypes that was greater than 5, and a number of DEGs in this group that was greater than 5. The resulting regulation model was consistent between the transcriptome and the qRT-PCR results (Figure 3), suggesting that our transcriptome analysis was reliable.

### 2.3. Global Analysis of DEGs

**Figure 4:**
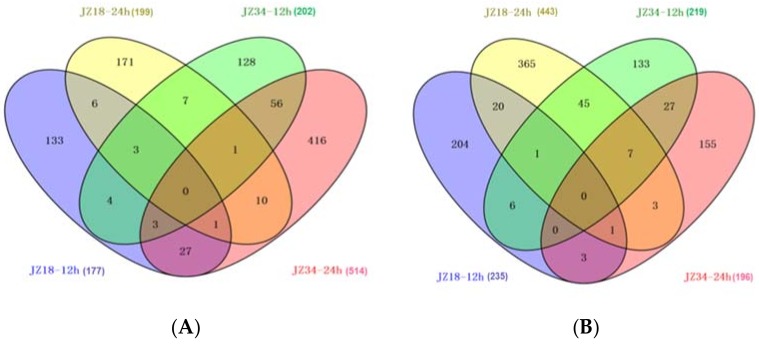
A Venn diagram showing overlaps among DEGs in *JZ18* and *JZ34*. The numbers of upregulated genes at 12 h and 24 h after K^+^-deficiency (0.5 mM) treatment (**A**); the numbers of downregulated genes at 12 h and 24 h after K^+^-deficiency (0.5 mM) treatment (**B**).

To investigate the molecular responses of the tomatoes under the K^+^-deficient condition, we identified up- and downregulated genes at 12 and 24 h post-treatment using the *t*-test (false discovery rate (FDR) ≤ 0.001) and an absolute value of log_2_ (fold change) ≥ 1 (treatment/control). We detected 1936 DEGs, including 966 upregulated and 970 downregulated genes. *JZ34* had more upregulated genes than did *JZ18*, whereas *JZ18* had more downregulated genes than did *JZ34*. The number of upregulated genes in *JZ34* was almost twice as high as that in *JZ18* (Figure 4). More DEGs were upregulated at 24 h than at 12 h. The gene expression patterns differed between *JZ34* and *JZ18*.

### 2.4. TFs in the DEGs

**Figure 5:**
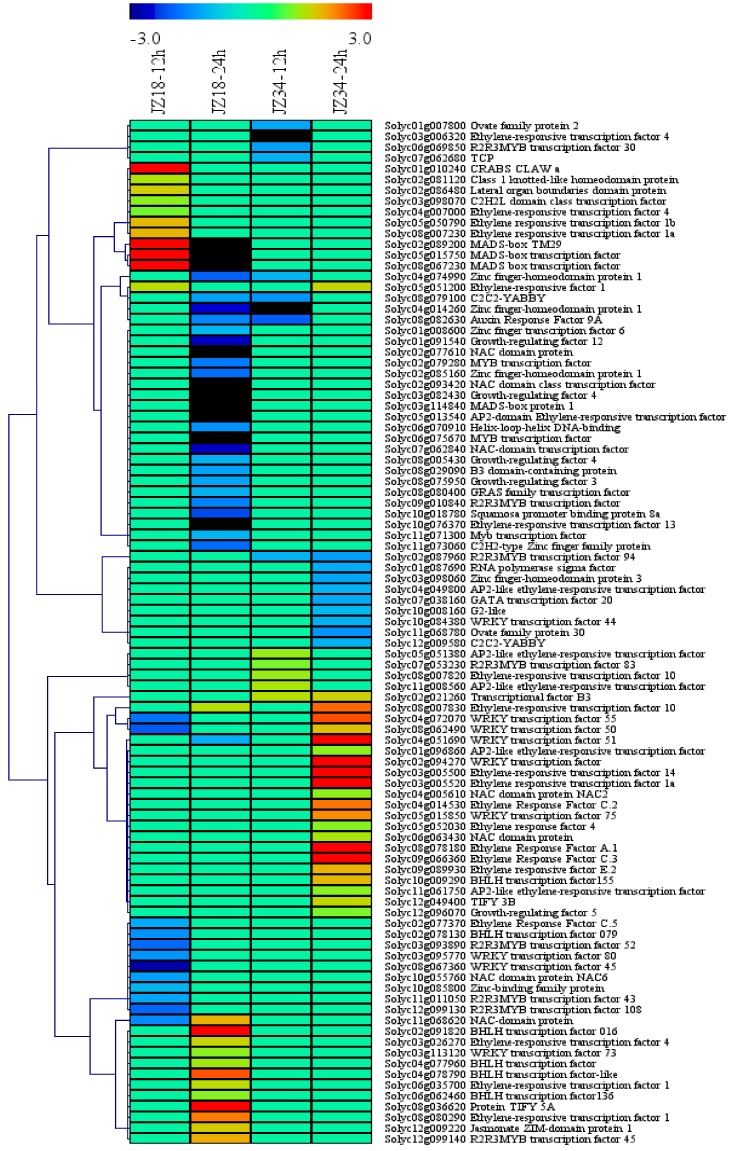
Average linkage hierarchical clustering analysis of transcription factors (TFs) identified among the DEGs.

Transcription factors (TFs) are essential for the regulation of gene expression in response to stress in higher plants. In this study, 95 DEGs encoding TFs were identified. These TFs belonged to diverse families (Figure 5), including ERF (19), MYB (11), WRKY (9), zinc finger (7), NAC (7), bHLH (6), GRF (5), MADS-box (4), AP2-EREBP (6), TIFY (2), OFP (2), C2C2-YABBY (2), ARF (1), B3 domain-containing protein (1), CRABS CLAW (1), G2-like (1), GATA (1), GRAS (1), C2H2L (1), class 1 knotted-like homeodomain protein (1), helix-loop-helix DNA-binding protein (1), jasmonate ZIM-domain protein 1 (1), lateral organ boundaries domain protein (1), RNA polymerase sigma factor (1), squamosal promoter binding protein (1), transcriptional factor B3 (1) and TCP(1). Among all of the TFs, the number of DEGs in the ERF and MYB families was greater than 10, accounting for up to 20% and 11.5% of the total TFs, respectively. All the TFs could be clustered into four categories. The *AP2*, *NAC*, *WRKY* and *GRF* genes were almost all downregulated in *JZ18* and upregulated in *JZ34*. *MYB*, *zinc finger*, *OFP* and *C2C2-YABBY* were all downregulated in both varieties, whereas *ERF*, *TIFY* and *bHLH* were all upregulated in both varieties. The *MADS* genes were upregulated at 12 h and downregulated at 24 h in *JZ18* but were not differentially expressed in *JZ34*.

### 2.5. DEGs Encoding Transporters and Kinases

Five genes encoding K^+^ channels and ion transporters demonstrated significantly differential expression in this study, including three upregulated K^+^ channel genes (*Solyc11g011500*, *Solyc03g097930*, and *Solyc01g010480*) and two downregulated K^+^ transporter genes (*Solyc12g009540* and *Solyc07g014680*). The expression of genes encoding nitrate and ammonium transporters changed greatly under K^+^-deficiency stress (Table 4). As the largest subfamily of receptor-like kinases (RLKs), leucine-rich repeat receptor-like kinases (LRR-RLKs) regulate the growth, development, and stress responses of plants. A total of 24 *LRR-RLK* DEGs were found, with 19 downregulated genes and two upregulated genes detected in *JZ18*. None downregulated genes and eight upregulated genes detected in *JZ34*. The overall expression trends of the *LRR-RLKs* in the two genotypes were opposite.

### 2.6. Differences in the Oxidative Stress, Hormone and Glycometabolism Analyses in JZ34JZ34 and JZ18 under K^+^ Deficiency

**Figure 6:**
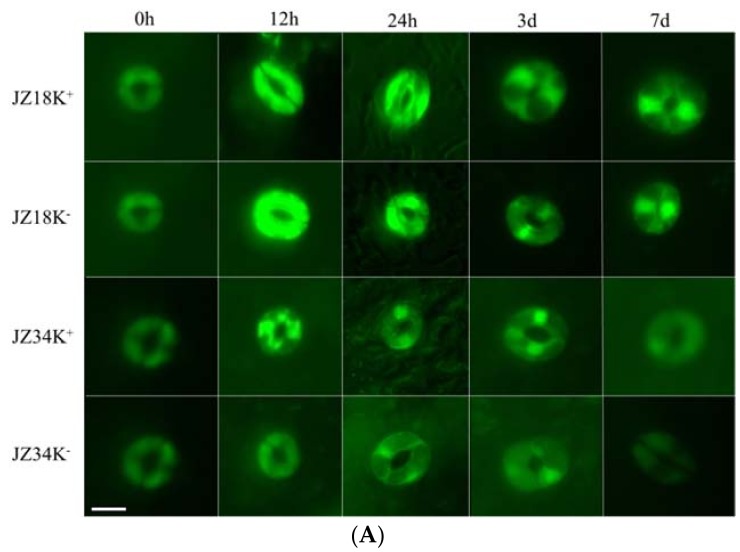

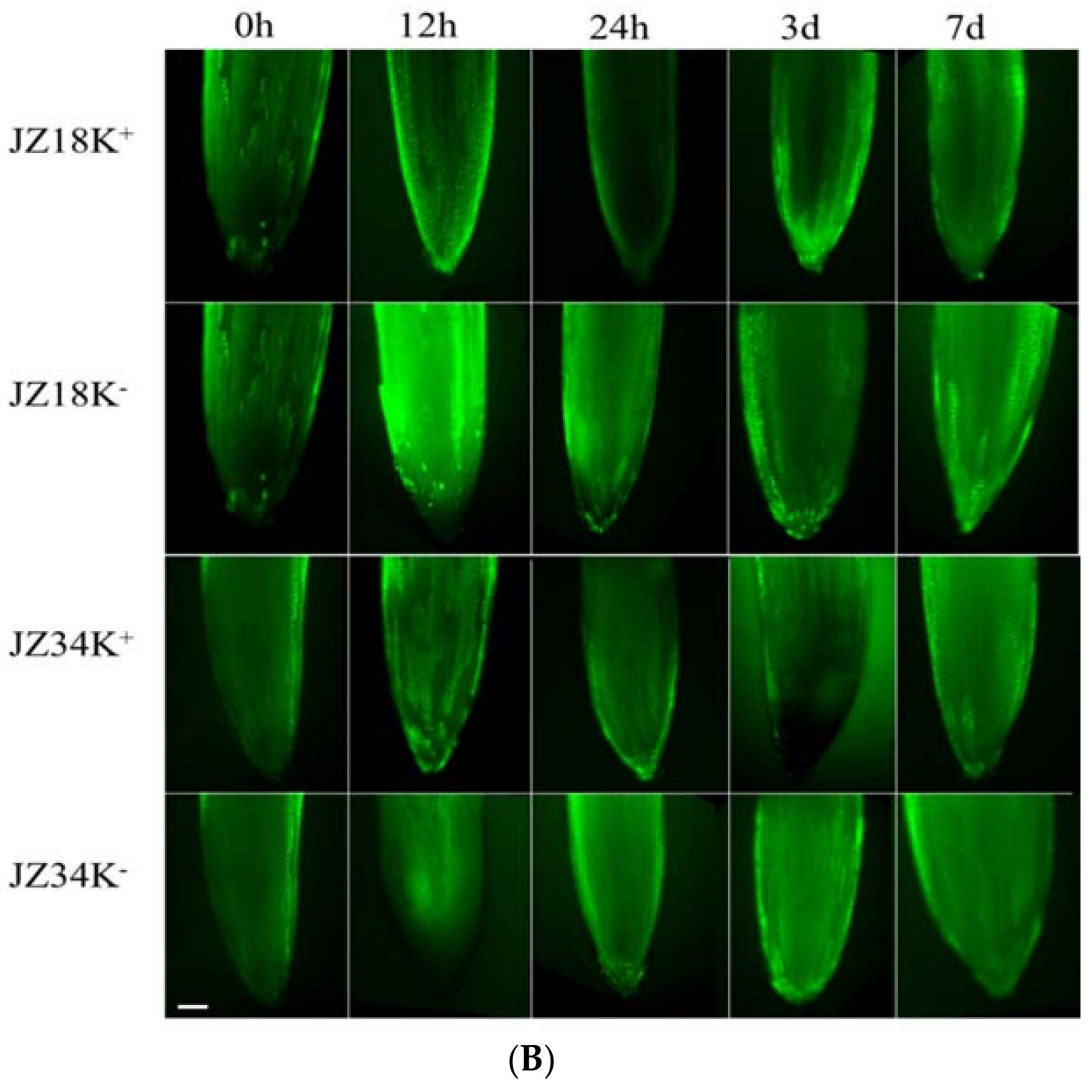
Comparison of the reactive oxygen species (ROS) content in the guard cells (**A**) and roots (**B**) of different tomato varieties under K^+^-deficiency stress conditions at 0 h, 12 h, 24 h, 3 d, and 7 d. K^+^ represents normal K^+^ (4 mM); K^−^ represents K^+^ deficiency (0.5 mM). The experiments were repeated three times. (**A**) Scale Bar: 10µm (**B**) Scale Bar: 30 µm.

The number of DEGs associated with oxidative stress metabolism was highest among all categories analyzed in the present study (Table S2). Among these genes, 42 peroxidase, 44 cytochrome P450, and 24 glutathione S-transferase genes were identified, including 46 upregulated and 56 downregulated genes. Approximately eight times as many genes were downregulated as upregulated in *JZ18*, whereas six times as many genes were upregulated as were downregulated in *JZ34*. The fluorescence signal was particularly strong in the guard cells and roots of *JZ18* (Figure 6). The determination of ROS concentrations in the plants revealed significant accumulation in the leaves and roots of *JZ18* at 12 h and 24 h (Table 5). These results show that stronger ROS scavenging activity is induced by K^+^-deficiency stress in *JZ34* than in *JZ18*.

**Figure 7:**
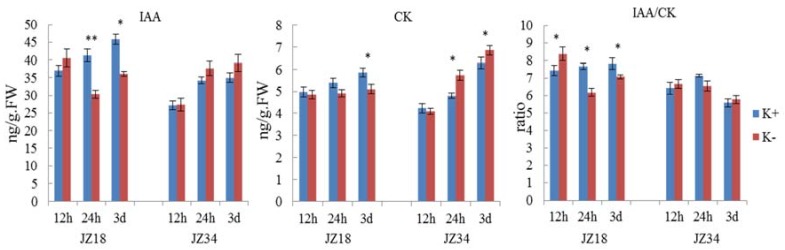
Comparison of the IAA and CK contents and the IAA/CK ratio in the roots of different tomato genotypes under K^+^-deficiency stress conditions at 12 h, 24 h, and 3 d. K^+^ represents normal K^+^ (4 mM); K^−^ represents K^+^ deficiency (0.5 mM). The experiments were repeated three times. * and ** denote significant differences at *p* < 0.05 and 0.01.

DEGs involved in hormone signaling were analyzed (Table 5), including auxin (5), gibberellin (12), cytokinin (8), ethylene (3), and salicylic acid (2). Genes encoding abscisic acid (ABA) -related genes were not found among the DEGs. Most DEGs were downregulated in *JZ18* and upregulated in *JZ34*. The number of auxin, gibberellin, and cytokinin genes was greater than five (Table 6). Plant hormones are very closely related to plant morphogenesis. To better explore the effects of phytohormones on tomato resistance under K^+^-deficiency and the effects on root architecture, the IAA and CK contents were determined and used to calculate the IAA/CK ratio under K^+^-deficiency (0.5 mM) treatment in the root systems of the two genotypes (Figure 7). The IAA content in the roots of the two genotypes was higher after K^+^-deficiency treatment than in the control at 12 h. The content in *JZ18* when the treatment was conducted for 24 h to 3 d was lower than that in the control group (Figure 7). At the same, the IAA content in *JZ34* was higher than that in the control group. These results showed that the IAA contents in the roots of the two genotypes were differentially altered after K^+^-deficiency stress. The CK content in the roots of the two genotypes showed an almost opposite trend after K^+^-deficiency stress, with lower levels in *JZ18* and higher levels in *JZ34* compared with those in the control group, except at 12 h. Typically, the IAA/CK ratio is used to determine whether to promote bud growth or root growth. A lower ratio enables promotion of bud differentiation, whereas a higher ratio is more conducive for root formation.

The IAA/CK ratio was higher in the treatment group than in the control group at 12 h in both genotypes. With extended treatment, the ratio was lower in the treatment group than in the control group at 24 h, and the difference was significant in *JZ18*. At 3 d of treatment, *JZ18* still showed a significantly lower ratio, whereas the *JZ34* treatment group had a higher ratio than did the control group.

In this study, 14 DEGs were associated with glycometabolism and transport. These genes included members of the SWEET family, as well as a glucose transporter, sucrose phosphate synthase, and ATP-dependent 6-phosphofructokinase. Of these DEGs, 11 were upregulated, and two were downregulated in *JZ34* (Table 7). Most of the genes related to glycometabolism were detected in *JZ34*, and almost all were upregulated. Although a few genes were detected in *JZ18*, most of these genes were downregulated.

### 2.7. DEGs Associated with Root Architecture

In the transcriptome analysis, five DEGs directly related to root architecture were found. All these DEGs were all upregulated in *JZ34* at 24 h (Table S3), with Solyc08g066590 was upregulated (2.31-fold) to the highest level compared to the expression of the other four genes.

### 2.8. GO and KEGG Analyses of K^+^-Deficiency Stress Tolerance-Related DEGs

**Figure 8:**
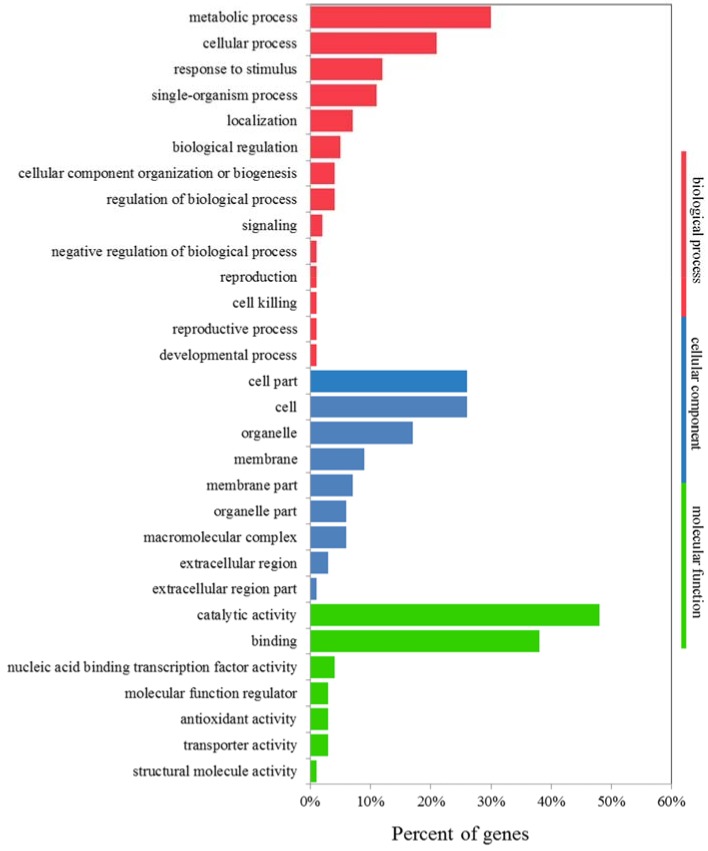
Gene Ontology (GO) for the co-expressed DEGs in the *JZ18* and *JZ34* genotypes.

Expression analysis by GO enrichment was conducted to identify DEGs that were significantly upregulated in *JZ34*, but downregulated or unchanged in *JZ18*, and DEGs that barely changed in *JZ34*, but were downregulated in *JZ18*. A total of 1210 DEGs met the above criteria under K^+^-deficiency stress in the two varieties (Figure S2 and Table S4). A total of 551 DEGs was downregulated in *JZ18* but unchanged in *JZ34*, whereas 517 DEGs were upregulated in *JZ34* but unchanged in *JZ18*. Only 161 DEGs that were found in both genotypes were upregulated in *JZ34* and downregulated in *JZ18*. More genes were differentially expressed at 24 h than at 12 h in the two accessions (Figure S2 and Table S4). The DEGs were divided into 36 functional groups. GO functional enrichment analysis showed that genes associated with binding (GO: 0005488) and catalytic activity (GO: 0003824) were significantly enriched, accounting for as much as 86% of the molecular function domain (Figure 8). The GO terms “metabolic process”, “cellular process”, “response to stimulus”, “single-organism process”, and “biological regulation” accounted for the majority of biological processes (Figure 8). The top two dominant terms in the cellular components were cell and cell part.

In total, 1210 DEGs encoding various enzymes were further matched with the enrichment of 117 KEGG pathways (Figure S3). The pathways with the greatest numbers of unique sequences were all metabolic pathways, including those related to secondary metabolites, amino acids, nucleotides, lipids, carbohydrates, energy, and other metabolism. The significantly enriched KEGG pathways are shown in Figure S3. The comparative transcriptome analysis of *JZ34* and *JZ18* under K^+^-deficiency stress laid a foundation for further elucidation of gene functions and the metabolic pathways in tomatoes.

## 3. Discussion

K^+^-deficiency stress in soil is quite common and becomes more severe during crop production [21]. Plants have evolved different strategies to cope with K^+^-deficiency. In the present study, a transcriptome analysis was performed 12 h and 24 h after K^+^-deficiency stress in two tomato genotypes. The early sequencing of adversity stress is more conducive to a comprehensive analysis of the differences in resistance caused by changes in the molecular mechanisms within the two genotypes. At the same time, we observed apparent differences in the root systems of the two genotypes after K^+^-deficiency treatment for 7 d. The experimental results proved that plants start from sensory signals, transmit the signals for self-regulation, change their phenotype, and finally adapt or not adapt to stress.

### 3.1. K^+^ Transporter Expression Results in Differences in Tolerance to K^+^ Deficiency between the Genotypes

K^+^ acquisition typically exhibits dual (high- and low-) affinity mechanisms in plants [9,22]. K^+^ uptake permeases (KUPs) encode plasma membrane K^+^/H^+^ symporters and catalyze K^+^ influx into cells under low-apoplastic-K^+^ conditions [23]. The transcript representing the KUP-related K^+^ transporter is *Solyc12g009540*; its expression was downregulated (6.79-fold) in *JZ18*, implying that this gene might assist the KUP gene family in K^+^ absorption under K^+^-deficient conditions. K^+^ translocation between different organs and tissues inside a plant and K^+^ secretion from root cortex cells into the xylem are mediated by outward-rectifying channels, such as SKOR [24]. *Solyc03g097930* is a SKOR-like K^+^ channel whose expression was upregulated in the low-K^+^-tolerant variety *JZ34*. *Solyc01g010480* is a voltage dependent K^+^-channel that is expressed in root hairs, and it displayed upregulated expression in *JZ34*. KAT1 is a highly selective inward-rectifying voltage-gated K^+^ channel that mediates long-term K^+^ influx into guard cells, leading to stomatal openings [25]. Expression of the *KAT1* gene transcript *Solyc01g010480* was upregulated (1.60-fold) in *JZ34*, suggesting that this variety was better able to perceive K^+^ deficiency, open stoma, and stimulate K^+^ transport. In our research, these genes were verified by qRT-PCR (Table S1).

### 3.2. Root-Related Genes, the ROS Signaling Pathway, and ERFs Cause Changes in Root Growth under K^+^ D Eficiency

Root morphological and physiological traits are important for the absorption of nutrients from the soil [26]. K^+^-efficient genotypes have a longer root system than do inefficient genotypes under K^+^-deficiency stress in rice [27], tomatoes [28], and *Hordeum maritimum* [29]. The differences in the root system of the two genotypes after K^+^-deficiency stress were very notable in our study. Similarly, K^+^ accumulation was positively correlated with the root length and root surface area in cotton [30]. Ethylene and ROS were also essential for root hair elongation [31]. Ethylene signaling plays an important role in low-K^+^-induced plant responses, such as root hair elongation, ROS production, and HAK5 expression [32].

Our study showed that the expression of many ERFs was upregulated, including *Solyc03g005520* (ERF1a) (8.16-fold) and *Solyc03g005500* (ERF14) (8.37-fold) (Table S1), were significantly higher in *JZ34* than in *JZ18* under the same conditions. The root fresh/ dry weight of *JZ34* was also higher than that of *JZ18*. Therefore, some ERFs may play an important role in root growth in tomatoes in response to low-K^+^ signaling in tomato. Furthermore, ethylene is known to act upstream of ROS in response to K^+^ deprivation in *Arabidopsis* [32]. K^+^-deprived plants rapidly accumulate ROS, and the expression of some K^+^ transporters is thought to be dependent on ROS production [33].

Peroxidase, cytochrome P450, and glutathione S-transferase are critical for the regulation of ROS production, and for reducing cellular damage during oxidative stress in plants [34]. In the present study, the highest number of DEGs was associated with oxidative metabolism than any other category. Whether or not this effect is related to ethylene needs further validation, but the oxidative stress response is clearly a key physiological activity in the response to short-term K^+^-deficiency stress. Our results showed differences in the ROS scavenging capacities of the two genotypes under K^+^-deficiency stress conditions, which was most likely a key factor underlying the different tolerances of these genotypes to low-K^+^. Peroxidase activity and ROS signaling are specifically required during LR emergence [35]. GPX was a peroxidase-encoding gene. In transcriptome analysis, *Solyc04g071890* (GPX1) and *Solyc07g056480* (GPX7) were upregulated (2.07-fold) in *JZ34* and downregulated (1.64-fold) in *JZ18*, respectively (Table S2). Overexpression of the two genes in *Arabidopsis* could enhanced root growth [36]. *Solyc07g052370* (CYP) is a cytochrome P450 gene that was upregulated (6.13-fold) (Table S1) in *JZ34* in the transcriptome analysis; this gene is involved in root defensive mechanisms in tomatoes [37]. Therefore, DEGs associated with oxidative stress are most likely key factors leading to differences in the root systems of the two genotypes under K^+^-deficiency stress.

### 3.3. K^+^ Deficiency between the Genotypes Leads to Changes in Hormonal Responses and Biosynthesis-Related DEGs

Plant hormones are continuously involved in the regulation of plant morphological changes under adverse conditions. Auxins play an important role in the development of plants, LRs, adventitious roots and root hairs. The PIN-FORMED (PIN) family serves as auxin export carrier proteins [38]. Many PIN family members participate in the transport and distribution of auxins, and thus play an important role in the establishment of auxin distribution patterns. The present study identified five auxin-related DEGs, four of which belong to the PIN family. *Solyc01g068410* (PIN5) was downregulated (2.65-fold) in *JZ18* and up-regulated (2.83-fold) in *JZ34*. Studies have suggested that PIN5 is mainly located in the endoplasmic reticulum; its expression in root hair cells can stimulate root hair growth to a certain extent, and PIN5 enhances the availability of internal IAA [39]. *Solyc10g080880* (PIN7) was downregulated in *JZ18*, and *Solyc07g006900* (PIN2) was upregulated in *JZ34*. Both genes are involved in IAA transport, and they regulate root growth and development [40]. This observation shows that genes associated with IAA transport are more active in *JZ34* than in *JZ18* and that the IAA content is increased in *JZ34* and is reduced in *JZ18* after K^+^-deficiency stress. Therefore, under K^+^-deficiency stress conditions, the PIN family showed differential expression patterns in the two genotypes, leading to differences in the IAA content to a certain extent, which might be one cause of the root system differences between the two genotypes. In addition, ROS have the ability to decrease IAA signaling [41], and therefore ROS accumulation in the roots of *JZ18* may inhibit IAA production and transport.

In our study, eight genes related to CK metabolism were identified. *Solyc10g017990*, (CKX3) was downregulated (7.65-fold) in *JZ18* (Table S1). In *Arabidopsis thaliana*, CKX3 promotes the growth of the main roots and LRs, and has a higher root biomass than does the wild type. Similar effects have also been found for CKX1, CKX2, and a family member of CKX in tobacco [42]. Therefore, we believe that DEGs that are associated with CKs are probably key factors leading to differences in the root systems of the two genotypes under K^+^-deficiency stress. We also measured the CK content. The *JZ18* CK content decreased after K^+^-deficiency stress, whereas that of *JZ34* increased. Existing studies suggest that CK has a negative regulatory effect on root growth and development [43]. However, the physiological processes of plants are often achieved by the coordinated regulation of various hormones. CKs can regulate the expression of auxin and PINs, and the ratio of IAA and CK can be used to explore synergistic on plant configurations more reasonably. Studies have suggested that a higher ratio indicates a greater utilization of root development. In terms of the IAA/CK ratio, *JZ34* gradually surpassed the control group at 3 d. At this time point, the root systems of the two genotypes began to change gradually in configuration (Figure 1). Notably, DEGs related to salicylic acid metabolism were also identified in the present study. Salicylic acid is a new type of plant hormone that regulates some important metabolic processes in plants [44]. Studies have shown that exogenous salicylic acid can induce increased growth in soybean roots [45] and act as a signaling molecule to induce sustained resistance to abiotic or abiotic stresses in various plants. Salicylic acid induces the production of many enzymes related to plant resistance, and regulates their activities [46]. After the initiation of K^+^-deficiency stress in the present study, salicylic acid related genes were downregulated in *JZ18* and upregulated in *JZ34*, with significant differences observed between the two genotypes. This hormone may be one key factor affecting the different tolerances of the two genotypes.

### 3.4. Glycometabolism and Transport Involved in K^+^-D Eficiency Stress

Sugar is not only is the energy source for plants, but also functions in signal transduction, regulates the expression and enzyme activity of related genes, and participates in the regulation of physiological activities [47,48]. Some studies have suggested that soluble sugars are involved in the production of NADPH, such as oxidation of the pentose phosphate pathway (OPP), to promote the clearance of ROS [49,50] and can also be used directly to scavenge hydroxyl radicals [51]. In the present study, the low-K^+^-tolerant strain showed no obvious ROS accumulation during the K^+^-deficiency treatment. This effect may be due to not only its strong ability to clear ROS, but also differences in sugar metabolism. SWEET family, glucose transporter, and solute carrier family genes involved in transporting sugar from the source to sink were also detected among the DEGs [52]. These DEGs were upregulated in *JZ34* and downregulated in *JZ18*. These genes promote the transport of sugar from the leaves to the roots, and provided a basis for root growth in *JZ34*; however, the sugar transport capacity was decreased, and root growth was inhibited in *JZ18* under K^+^-deficiency stress. Of these DEGs, *Solyc03g097560* (SWEET14) was most downregulated (6.82-fold) in *JZ18* (Table S1), which include *cis*-acting elements that respond to auxin. Thus, this gene is very likely to have a negative impact on IAA production. Based on our analysis, sugar metabolism and transport-related genes appear to play a positive role in improving plant resistance to K^+^-deficiency stress. Genes directly related to root development were also screened by the transcriptome analysis, but no further studies were found for these genes in tomatoes. Among the 20 genes identified in this study, the genes discussed include ERF, PIN, CKX, CYP, and SWEET14, which we believe were more likely to have an effect on root configuration under low K^+^ stress, and could provide a good basis for subsequent in-depth experiments.

**Figure 9:**
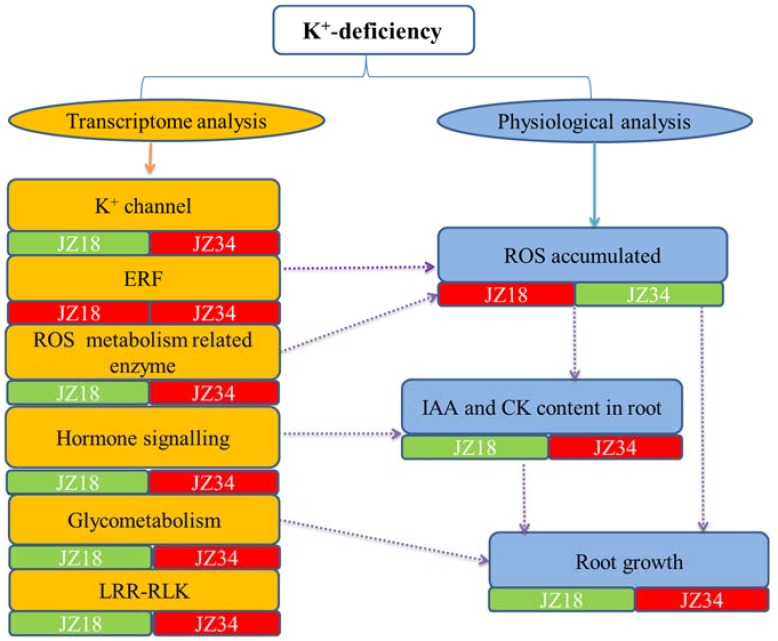
A hypothetical model of the response to K^+^-deficiency stress in two tomato genotypes. The green box represents downregulated DEGs, and the red box represents upregulated DEGs. The dotted lines represent potential relationships.

Two tomato genotypes were exposed to short-term K^+^-deficiency stress in this work. Multiple physiological metabolic pathways are involved in responses to K^+^-deficiency stress (Figure 9). Combining the transcriptome analysis and physiological analysis results suggested that a potential interaction occurred between them. ERF and ROS metabolism-related enzymes are probably involved in ROS accumulation and clearance. Hormone signaling is instructive for changes in the IAA and CK contents. Glycometabolism may play a very positive role in changes in the root architecture. No investigation found that LRR-RLKs affected the root architecture under K^+^-deficiency stress conditions, but these results indicated that LRR-RLKs might be more important in the tomato response to K^+^-deficiency. The changes in the ROS, IAA, and CK contents at the physiological level explained the changes in root architecture, although the mechanism underlying K^+^-deficiency stress resistance requires further study. In addition, the mechanisms regulating K^+^ absorption, transport, and utilization are important for tomato root system growth and plant yields with the increasingly widespread issue of K^+^-deficient soil. Our current study has provided valuable materials and data for further research into K^+^-deficiency stress.

## 4. Materials and Methods

### 4.1. Plant Materials and K^+^ Deficiency

Seeds of two tomato genotypes (*JZ34*, low-K^+^-tolerant, and *JZ18*, low-K^+^-sensitive) were surface sterilized in 1.0 mL 10% (*v*/*v*) NaClO for 15 min and rinsed five times with distilled water. The seeds were incubated to allow them to germinate in plastic Petri dishes in the dark for 3 d at 25 °C on two layers of sterile filter paper that were previously soaked in water. Tomato seedlings were transferred to a tray containing a 1:1:1 (*v*/*v*/*v*) mix of peat: perlite: vermiculite and grown in a greenhouse from March to April with day/night temperatures of approximately 26/18 °C in a daily average irradiance of 350 μmol·m^−2^·s^−1^ and at 70% relative humidity (RH). Seedlings at the vegetative growing stage (25 d) were cleaned with water, washed three times with distilled water and transferred to a pot (50 cm length, 30 cm width, 10 cm height) containing 12 L of nutrient solution. Each pot was filled with nutrient solution containing 1.5 mM Ca(NO_3_)_2_·4H_2_O, 4 mM KNO_3_, 0.67 mM NH_4_H_2_PO_4_, 2 mM MgSO_4_·7H_2_O, 0.05 mM H_3_BO_3_, 0.009 mM MnSO_4_·4H_2_O, 0.7 mM ZnSO_4_·7H_2_O, 0.32 mM CuSO_4_·5H_2_O, 0.1 mM (NH_4_)_2_MoO_4_, 0.05 mM FeSO_4_·7H_2_O, and 0.04 mM Na_2_-EDTA. The pH was adjusted to 5.8 ± 0.1 as required. The nutrient solution was replaced once every 3 d. A total 24 pots were used, with each pot containing nine plants. At the vegetative growing stage (30 d), K^+^ deficiency treatment was applied by reducing the concentration of KNO_3_ from 4 mM (normal K^+^) to 0.5 mM (K^+^ deficiency) in the nutrient solution. A concentration of 4 mM KNO_3_ was used as a control. After 7 d of K^+^-deficiency stress, different parts of the plant were sampled and dried to assess plant length, dry weight, and K^+^ content. K^+^ accumulation = K^+^ content × dry weight; K^+^ use efficiency (KUE) = dry weight (g) × 1000/K^+^ content (mg/g). To more clearly observe the apparent difference between the two genotypes after K^+^-deficiency stress, K^+^-free nutrient solution was also used. Differences in the leaf (Figure S1) and root systems of the two genotypes were examined at 3 d and 7 d, respectively. The experiments were repeated at least three times.

### 4.2. Measurement of K^+^ Uptake Kinetics

The nutrient solution of four-leaf-stage (25 day) tomato seedlings was replaced with a solution without K^+^ for 3 d with day/night temperatures of approximately 26/18 °C. Three seedlings with consistent growth were washed three times with 0.2 mM CaSO_4_ and placed into a black triangular flask with 100 mL of absorption liquid (0.2 mM KCl + 0.2 mM CaSO_4_). This process was repeated three times with each strain. Next, 1 mL of absorption liquid was removed from the triangular flask at different time points, and 1 mL of distilled water was added; this process was repeated until the absorption liquid K^+^ concentration was stable or close to zero. The K^+^ uptake kinetic parameters (K_m_ and I_max_) were calculated according to the method of Drew et al. [53]. I_max_ is the maximum uptake rate, and K_m_ is the Michaelis-Menten constant.

### 4.3. RNA-seq Sampling and RNA Isolation

Seeds of *JZ34* and *JZ18* were germinated for RNA-seq sampling under the conditions described above and placed into a plant growth chamber. Four-leaf-stage seedlings at the vegetative growth stage (30 d) were exposed to K^+^-deficiency stress (0.5 mM) or the full-strength solution as control for 12 h and 24 h. Three whole independent seedlings were collected and mixed together at each time point to reduce the differences between individual plants. Three biological replicates were included for each treatment. The samples were snap-frozen in liquid nitrogen and stored at −80 °C for the RNA-seq analysis.

RNA was isolated according to the instructions of the RNeasy mini kit (Qiagen, Hilden, Germany). The RNA purity was determined using the NanoPhotometer^®^ spectrophotometer (IMPLEN, Westlake Village, CA, USA). The RNA concentration was measured using the Qubit^®^ RNA Assay Kit in the Qubit^®^ 2.0 Fluorometer (Life Technologies, Carlsbad, CA, USA). The RNA integrity number (RIN) was assessed using the RNA Nano 6000 Assay Kit on the Agilent Bioanalyzer 2100 system (Agilent Technologies, Santa Clara, CA, USA) with 2.2 ≥ A260/A280 ≥ 2.0, 28 S/*JZ18* S ≥ 1.5 and RIN ≥ 7.5. The RNA abundances and purity were tested to confirm that they met our requirements.

### 4.4. Library Construction, Sequencing, and Data Processing

Library preparations were sequenced on the BGISEQ-500 platform (BGI, Shenzhen, China), and 50-bp single-end (SE) reads were generated. BGISEQ-500 is powered by combinatorial probe-anchor synthesis (cPAS) and improved DNA Nanoballs (DNBs) technology. The cPAS chemistry works by incorporating a fluorescent probe into a DNA anchor on the DNBs, followed by high-resolution digital imaging. This combination of linear amplification and DNB technology reduces the error rate while enhancing the signal. In addition, the size of the DNBs is controlled so that only one DNB is bound per active site. This patterned array technology not only provides sequencing accuracy but also increases chip utilization and sample density.

### 4.5. Identification of DEGs

High-quality clean reads were mapped to the reference transcriptome, and the transcript levels of the unigenes were identified by TopHat (http://ccb.jhu.edu/software/tophat/index.shtml) and Cufflinks (http://cole-trapnell-lab.github.io/cufflinks/) [54] and normalized by the Fragments Per Kilobase of exon model per million mapped reads (FPKM) approach [55] according to the following formula: FPKM = 10^6^C/NL/10^3^. Given the expression of gene A, C indicates the number of fragments aligned to gene A, N represents the total number of fragments aligned to all genes, and L denotes the number of bases in gene A.

Sequencing was used to identify genes that were differentially expressed in the two different varieties [56]. FDRs were used in multiple hypothesis testing to correct the *p*-values. The FDRs were statistically preset to values less than 0.05, and the differential gene expression in different samples was calculated based on the FPKM values [57]. The expression ratio of 12 h/0 h or 24 h/0 h is presented as the fold change in the present study. An FDR ≤ 0.001 and an absolute value of log_2_ (fold-change) ≥ 1 were used as the thresholds to screen DEGs. The Multiexperiment Viewer (MeV; http://www.tm4.org/mev.html) [58] was used to delineate heatmaps based on the DEG results.

### 4.6. Gene Annotation, GO Enrichment and KEGG Analysis

The GO and pathway enrichment analyses of the DEGs were performed using the PANTHER classification system (http://www.pantherdb.org/data/) [59] and KEGG (http://www.kegg.jp/) [60]. A basic local alignment search tool (BLASTn) programme was used to identify homologues in the tomato genome (https://solgenomics.net/).

### 4.7. qRT-PCR Analysis

For quantitative qRT-PCR analysis, first-strand cDNA was synthesized using the PrimeScript 1st Strand cDNA Synthesis Kit (TIANGEN, Beijing, China). The qRT-PCR was performed in triplicate for each sample using the SYBR Green Real Master Mix, according to the manufacturer’s instructions. qRT-PCR amplification was performed using the Bio-Rad CFX Manager 3.1 Real Time PCR System (Applied Biosystems, Foster City, CA, USA) and software (Applied Biosystems). The 2^−∆∆*C*t^ method was used to analyze the relative changes in the gene expression levels from three biological replicates. The data were analyzed based on the mean values of triplicates. The specificity of the reactions was verified through melting-curve analysis. *Actin* was used as an internal control. The primers used for the qRT-PCR are listed in Table S5.

### 4.8. Determination of ROS

The cultivation method was the same as described above, and K^+^-deficiency treatment was used to detect ROS in tomato leaves and roots at 0, 12 h, 24 h, 3 d, and 7 d. Three biological replicates were conducted for each treatment. ROS accumulation was detected using the Reactive Oxygen Species Assay kit (Beyotime Biotechnology Shanghai, Shanghai, China) on the ZEISS Axio Observer A1 inverted fluorescence microscope. The H_2_O_2_ and O_2_^−^ concentrations in the leaves and roots were determined according to a modified method [61,62].

### 4.9. Endogenous Hormone Content Determination

At 12 h, 24 h, and 3 d of K^+^-deficiency treatment, 0.1 g of root material was collected from each genotype. The experiments were repeated at least three times. The hormone contents were analyzed using an enzyme-linked immunosorbent assay [63].

### 4.10. Statistical Analysis

The data were analyzed using one-way analysis of variance (ANOVA) followed by Tukey’s honestly significant difference (HSD) multiple comparison tests in Statistical Productions and Service Solutions 17.0 (SPSS, Chicago, IL, USA). *p* < 0.05 was considered significant. Asterisks (* and **) indicate a significant difference between the controls and transgenic plants at *p* < 0.05 and 0.01, respectively.

## Figures and Tables

**Table 1:** Effect of K^+^ level on the biomass, K^+^ concentration and accumulation in two different genotypes at 7 d.

	Genotype	K^+^	K^−^	Relative Index
Stem length (mm)	*JZ34*	36.33a	34.67a	0.95
	*JZ18*	28.67a	24.00a	0.84
Root length (mm)	*JZ34*	21.67a	21.50a	0.99
	*JZ18*	25.00a	21.50b	0.86
Fresh weight (mg·plant^−1^ FW)	*JZ34*	12.08a	12.44a	1.03
	*JZ18*	10.46a	10.01a	0.96
Dry weight (mg·plant^−1^ DW)	*JZ34*	0.79a	0.85a	1.07
	*JZ18*	0.77a	0.55b	0.71
K^+^ content (mg·g^−1^ DW)	*JZ34*	367.00a	325.71a	0.89
	*JZ18*	389.88a	223. 34b	0.57
K accumulation (mg·plant^−1^)	*JZ34*	289.20a	276.53a	0.95
	*JZ18*	286.17A	129.98B	0.45

The experiments were repeated three times. K^+^ represents normal K^+^ (4 mM); K^−^ represents K^+^ deficiency (0.5 mM). For each line, different lowercase and uppercase letters indicate significant differences (*p* < 0.05 and *p* < 0.01 respectively) among the treatments.

**Table 2:** Overview of the digital gene expression profiling of the sequencing data.

Sample	Raw Data Size (nt)	Raw Reads Number	Clean Data Size	Clean Reads Number	Clean Data Rate (%)	Total Mapped Reads (%)	Q20 (%)
*JZ18*-ck12h	1206807650	24136153	1204621000	24092420	99.81	80.53	96.7
*JZ18*-lk12h	1206819150	24136383	1201320750	24026415	99.54	81.54	96.9
*JZ18*-ck24h	1206831700	24136634	1202634100	24052682	99.65	81.69	96.7
*JZ18*-lk24h	1206825700	24136514	1202777750	24055555	99.66	80.22	96.9
*JZ34*-ck12h	1206841700	24136834	1176860650	23537213	97.51	81.98	97.0
*JZ34*-lk12h	1206833100	24136662	1199255700	23985114	99.37	80.78	97.5
*JZ34*-ck24h	1206848550	24136971	1203941400	24078828	99.75	83.08	97.6
*JZ34*-lk24h	1206808900	24136178	1204460950	24089219	99.8	83.75	97.6

**Table 3:** Expression levels of the housekeeping control genes at 12 h and 24 h.

Housekeeping Gene	Gene ID	Treatment Time (h)	*JZ18* Log2 Fold Change	FDR	Significant	*JZ34* Log2 Fold Change	FDR	Significant
*GAPDH*	*Solyc04g009030.2*	12	0.01	1.00	no	0.02	1.00	no
		24	0.03	1.00	no	0.02	1.00	no
*GAPDH*	*Solyc12g094640.1*	12	−0.10	1.00	no	0.00	0.99	no
		24	0.81	1.00	no	−0.76	1.00	no
*Catalase*	*Solyc02g082760.2*	12	0.13	1.00	no	0.13	1.00	no
		24	0.14	1.00	no	0.05	0.07	no
*Catalase*	*Solyc12g094620.1*	12	0.49	1.00	no	−0.27	1.00	no
		24	0.72	1.00	no	0.21	1.00	no
*Cys protease*	*Solyc08g082400.1*	12	0.05	0.83	no	−0.14	0.15	no
		24	0.15	0.24	no	−0.31	1.00	no
*Cys protease*	*Solyc12g095910.1*	12	0.24	1.00	no	−0.12	1.00	no
		24	0.04	0.70	no	0.26	1.00	no
*α-Tubulin*	*Solyc04g077020.2*	12	0.15	1.00	no	−0.03	0.23	no
		24	−0.06	0.11	no	−0.04	0.12	no
*Ubiquitin*	*Solyc07g064130.1*	12	−0.16	1.00	no	0.00	0.86	no
		24	−0.05	1.00	no	−0.22	1.00	no
*Actin*	*Solyc04g011500.2*	12	0.04	0.75	no	0.13	0.19	no
		24	−0.07	0.27	no	0.05	0.41	no
*DNAJ chaperone*	*Solyc11g006460.1*	12	0.19	1.00	no	0.04	0.25	no
		24	0.03	0.36	no	−0.02	0.71	no
*Translation initiation factor 5A*	*Solyc12g010060.1*	12	0.12	1.00	no	0.11	1.00	no
		24	0.01	0.87	no	0.24	1.00	no

**Table 4:** Genes encoding protein transporters and kinases showed genotypic differences in response to K^+^-deficiency (0.5 mM) stress.

Group	Gene ID	log_2_ (Fold Change)				Seq Description
		*JZ18*		*JZ34*		
		12 h	24 h	12 h	24 h	
potassium	*Solyc11g011500*	1.30				Potassium channel
	*Solyc03g097930*			1.06		potassium channel SKOR-like
	*Solyc01g010480*				1.60	Potassium voltage-gated channel
	*Solyc12g009540*	−6.79				KUP system potassium uptake protein
	*Solyc07g014680*				−1.25	Potassium transporter
Nitrate	*Solyc11g069760*	1.36	−2.69			High affinity nitrate transporter protein
	*Solyc08g007430*	1.23				Nitrate transporter
	*Solyc06g010250*	−2.20	−1.85			Nitrate transporter
	*Solyc00g090860*	1.21	−1.83			Nitrate transporter
	*Solyc07g032490*		1.10			Nitrate transporter
	*Solyc08g078950*		−1.18			Nitrate transporter
	*Solyc08g007430*		−1.23			Nitrate transporter
	*Solyc11g069750*			1.70		Nitrate transporter
	*Solyc03g113250*				−1.15	Nitrate transporter
	*Solyc11g069740*				−1.56	Nitrate transporter
Yellow-strike	*Solyc03g082620*				2.10	Metal-nicotianamine transporter
Mate	*Solyc07g006740*	1.63				MATE efflux family protein
	*Solyc11g065820*				1.13	MATE efflux family protein
	*Solyc03g026230*		1.57			MATE efflux family protein
	*Solyc08g080310*	1.12	−1.32			MATE efflux family protein
LRR	*Solyc01g005870*				1.74	LRR receptor-like protein kinase
	*Solyc06g033920*	−1.01				LRR receptor-like protein kinase
	*Solyc01g009810*	−2.04				LRR receptor-like protein kinase
	*Solyc01g091230*		−1.20			LRR receptor-like protein kinase
	*Solyc01g098690*		1.56			LRR receptor-like protein kinase
	*Solyc02g071820*				1.57	LRR receptor-like protein kinase
	*Solyc03g005960*		−1.65			LRR receptor-like protein kinase
	*Solyc03g007050*		−1.41			LRR receptor-like protein kinase
	*Solyc04g014400*	−1.16			1.74	LRR receptor-like protein kinase
	*Solyc04g014900*	−1.06				LRR receptor-like protein kinase
	*Solyc04g054450*	−1.66				LRR receptor-like protein kinase
	*Solyc04g074000*	−1.10	−1.97		3.67	LRR receptor-like protein kinase
	*Solyc04g074020*		−1.73			LRR receptor-like protein kinase
	*Solyc04g074030*		−3.12		5.49	LRR receptor-like protein kinase
	*Solyc04g074050*		−2.08		2.11	LRR receptor-like protein kinase
	*Solyc04g081080*		−1.10			LRR receptor-like protein kinase
	*Solyc06g006020*	−2.89	−1.23			LRR receptor-like protein kinase
	*Solyc09g014480*	1.14				LRR receptor-like protein kinase
	*Solyc09g082530*	−1.15				LRR receptor-like protein kinase
	*Solyc10g007830*		−3.32			LRR receptor-like protein kinase
	*Solyc12g009730*	−1.43				LRR receptor-like protein kinase
	*Solyc12g009750*	−1.43				LRR receptor-like protein kinase
	*Solyc12g009780*	−3.56			2.25	LRR receptor-like protein kinase
	*Solyc11g011180*				2.83	LRR receptor-like protein kinase
CRK	*Solyc01g006390*	2.60	−5.03			Cysteine-rich receptor-like protein kinase
	*Solyc03g111530*		−4.90			Cysteine-rich receptor-like protein kinase
	*Solyc11g006430*		−2.52	−1.29		Cysteine-rich receptor-like protein kinase
	*Solyc07g005110*			1.34		Cysteine-rich receptor-like protein kinase
	*Solyc05g018930*				−2.59	Cysteine-rich receptor-like protein kinase

**Table 5:** H_2_O_2_ and O_2_^−^ levels in the leaves and roots of *JZ18* and *JZ34* under K^+^-deficiency stress conditions at 0 h, 12 h, 24 h, 3 d, and 7 d. K^+^ represents normal K^+^ (4 mM); K^−^ represents K^+^ deficiency (0.5 mM). The experiments were repeated three times.

	H_2_O_2_ Concentration/nmol·min^−1^·g^−1^ FW				O_2_^− ^Concentration/nmol·min^−1^·g^−1^ FW			
	*JZ18*K^+^	*JZ18*K^−^	*JZ34*K^+^	*JZ34*K^−^	*JZ18*K^+^	*JZ18*K^−^	*JZ34*K^+^	*JZ34*K^−^
	Leaf							
0 h	31.07 ± 1.73	30.66 ± 2.35	19.02 ± 1.42	19.33 ± 1.43	3.91 ± 0.06	3.89 ± 0.22	3.07 ± 0.05	3.21 ± 0.25
12 h	30.88 ± 1.09	40.10 ± 2.07 **	22.00 ± 1.47	22.66 ± 1.67	3.80 ± 0.14	4.72 ± 0.15 *	2.88 ± 0.14	2.87 ± 0.24
24 h	31.01 ± 1.33	33.40 ± 2.31	21.16 ± 2.00	23.97 ± 2.05	3.49 ± 0.21	4.43 ± 0.20 *	2.66 ± 0.04	2.46 ± 0.12
3 d	32.00 ± 1.68	36.73 ± 0.66	18.62 ± 1.01	18.12 ± 2.01	3.58 ± 0.24	4.14 ± 0.24	2.78 ± 0.17	2.44 ± 0.10
7 d	31.58 ± 0.77	34.95 ± 2.55	19.45 ± 1.65	19.11 ± 2.23	3.70 ± 0.20	4.38 ± 0.12	2.87 ± 0.15	2.70 ± 0.17
	Root							
0 h	6.45 ± 0.17	6.62 ± 0.13	4.42 ± 0.21	4.44 ± 0.04	0.96 ± 0.03	0.88 ± 0.04	0.83 ± 0.03	0.84 ± 0.02
12 h	5.98 ± 0.25	8.79 ± 0.25 **	4.86 ± 0.23	4.25 ± 0.14	0.71 ± 0.03	1.06 ± 0.04 **	0.77 ± 0.05	0.70 ± 0.04
24 h	6.66 ± 0.18	7.75 ± 0.16 *	3.66 ± 0.16	4.07 ± 0.13	0.66 ± 0.04	0.73 ± 0.01	0.75 ± 0.02	0.87 ± 0.01 *
3 d	6.10 ± 0.23	6.29 ± 0.09	3.78 ± 0.09	3.65 ± 0.16	0.70 ± 0.02	0.75 ± 0.03	0.71 ± 0.01	0.74 ± 0.03
7 d	6.98 ± 0.03	7.24 ± 0.11	3.62 ± 0.19	3.76 ± 0.03	0.74 ± 0.04	0.77 ± 0.03	0.73 ± 0.02	0.66 ± 0.01

* and ** denote significant differences at *p* < 0.05 and 0.01, respectively.

**Table 6:** Folds change of DEGs related to hormone signaling in response to K^+^-deficiency (0.5 mM) stress.

Transcript ID	*JZ18*-12 h	*JZ18*-24 h	*JZ34*-12 h	*JZ34*-24 h	Seq Description
*Solyc01g068410*	−2.65		2.83		Auxin Efflux Carrier
*Solyc10g080880*		−1.00			Auxin Efflux Carrier
*Solyc04g056620*			2.11		Auxin Efflux Carrier
*Solyc07g006900*			1.14	1.13	Auxin Efflux Carrier
*Solyc02g082450*			−1.63		Auxin Efflux Carrier
*Solyc12g042980*	1.83				Gibberellin 20-oxidase
*Solyc03g006880*	1.14				Gibberellin 20-oxidase-1
*Solyc11g072310*			1.55		Gibberellin 20-oxidase-3
*Solyc03g025490*			1.44		Gibberellin 20-oxidase-like protein
*Solyc06g066820*		−1.51			Gibberellin 3-beta-hydroxylase
*Solyc07g056670*		1.74			Gibberellin 3-oxidase
Solyc12g042980		−3.27			Gibberellin 3-beta-dioxygenase
*Solyc00g138060*			1.91	1.35	Gibberellin 2-oxidase
*Solyc01g090630*	−1.04				Gibberellin 2-oxidase
*Solyc01g079200*		2.57			Gibberellin 2-oxidase
*Solyc02g080120*			1.36	1.89	Gibberellin 2-beta-dioxygenase
*Solyc02g070430*				1.11	Gibberellin 2-oxidase 1
*Solyc06g076550*	−1.54				Cytokinin-N-glucosyltransferase
*Solyc04g074380*		−2.12			Cytokinin-N-glucosyltransferase
*Solyc10g085280*				1.18	Cytokinin-N-glucosyltransferase
*Solyc12g057080*				−1.39	Cytokinin-N-glucosyltransferase
*Solyc12g008900*		−4.91			Cytokinin dehydrogenase 1
*Solyc10g017990*		−7.65			Cytokinin dehydrogenase 1
*Solyc02g079440*			1.47	1.04	Cytokinin dehydrogenase
*Solyc06g082730*				1.42	Cytokinin trans-hydroxylase
*Solyc09g089580*	−1.49		−1.31		Ethylene metabolic process
*Solyc02g091990*		2.15			Ethylene metabolic process
*Solyc07g049530*		−1.00			Ethylene metabolic process
*Solyc09g091550*	−2.9		2.44		Salicylic acid carboxyl methyltransferase
*Solyc09g091540*				6.92	Salicylic acid carboxyl methyltransferase

**Table 7:** Fold changes of DEGs related to glycometabolism in response to K^+^-deficiency (0.5 mM) stress.

Transcript ID	*JZ18*-12 h	*JZ18*-24 h	*JZ34*-12 h	*JZ34*-24 h	Seq Description
*Solyc03g097560*		−6.82			bidirectional sugar transporter SWEET14
*Solyc03g097600*			1.40	1.69	bidirectional sugar transporter SWEET10-like
*Solyc06g072620*			1.24		bidirectional sugar transporter SWEET10-like
*Solyc03g097610*			1.22		bidirectional sugar transporter SWEET10
*Solyc06g060590*				1.41	bidirectional sugar transporter SWEET1
*Solyc01g080680*			1.01	1.04	Glucose transporter 8
*Solyc06g054270*				1.05	Solute carrier family 2 Sugar/inositol transporter
*Solyc09g075820*				1.81	Solute carrier family 2 Sugar/inositol transporter
*Solyc11g067340*				1.02	Acyltransferase (Fragment)
*Solyc09g092130*		1.24		−1.58	Sucrose phosphate synthase
*Solyc08g066100*				6.22	ATP-dependent 6-phosphofructokinase
*Solyc12g009300*				2.68	Sucrose synthase
*Solyc08g079080*		−1.33		1.17	vacuolar invertase, Lin9
*Solyc02g086530*			−3.08		Alpha-galactosidase

## Supplementary Materials

Supplementary materials can be found at http://www.mdpi.com/1422-0067/19/8/2402/s1.

## Abbreviations

ABA	Abscisic acid
AP2/ERF	Apetala 2 DNA binding domain transcription factor/ethylene-responsive factor
ARF	Auxin response factor
BGISEQ-500	Big sequencing system-500
bHLH	Basic helix-loop-helix
BLAST	Basic local alignment search tool
cPAS	Combinatorial probe-anchor synthesis
CK	Cytokinin
CKX	Cytokinin oxidase genes
CPA	Cation proton antiporters
DEGs	Differentially expressed genes
DNB	DNA nanoballs
ERF	Ethylene-responsive factor
EDTA	Ethylenediamine tetraacetic acid
FDR	False discovery rate
FPKM	Fragments per kilobase of exon model per million mapped reads
GO	Gene ontology
GA	Gibberellins
GAPDH	Glyceraldehyde-3-phosphate dehydrogenase
GPX	Glutathione peroxidase
IAA	Auxin
I_max_	Ion maximum absorption rate
K_m_	Michaelis-Menten constant
KEGG	Kyoto encyclopedia of genes and genomes
KUE	K use efficiency
LR	Lateral root
LRR-RLKs	Leucine-rich repeat receptor-like protein kinases
GRF	Growth-regulating factor genes
OFP	Ovate family proteins
RH	Relative humidity
PIN	PIN-formed
ROS	Reactive oxygen species
SWEET	Sugars will eventually be exported transporter
SKOR	Stelar K^+^ outward rectifying channel
TFs	Transcription factors.
