# Supplementary material for: Comparative Transcriptome Profiling of Two Tomato Genotypes in Response to Potassium-Deficiency Stress
Source: Int J Mol Sci. 2018 Aug 14;19(8):2402. doi: 10.3390/ijms19082402 (PMC6121555; doi:10.3390/ijms19082402)
Supplement: Supplementary file 1 [file ijms-19-02402-s001.zip › ijms-323017 supplementary update/Figure S1.pdf]

Figure S1: Comparison of the leaf changes in different tomato varieties under K^+-deficiency stress conditions at 3 d and 7 d.

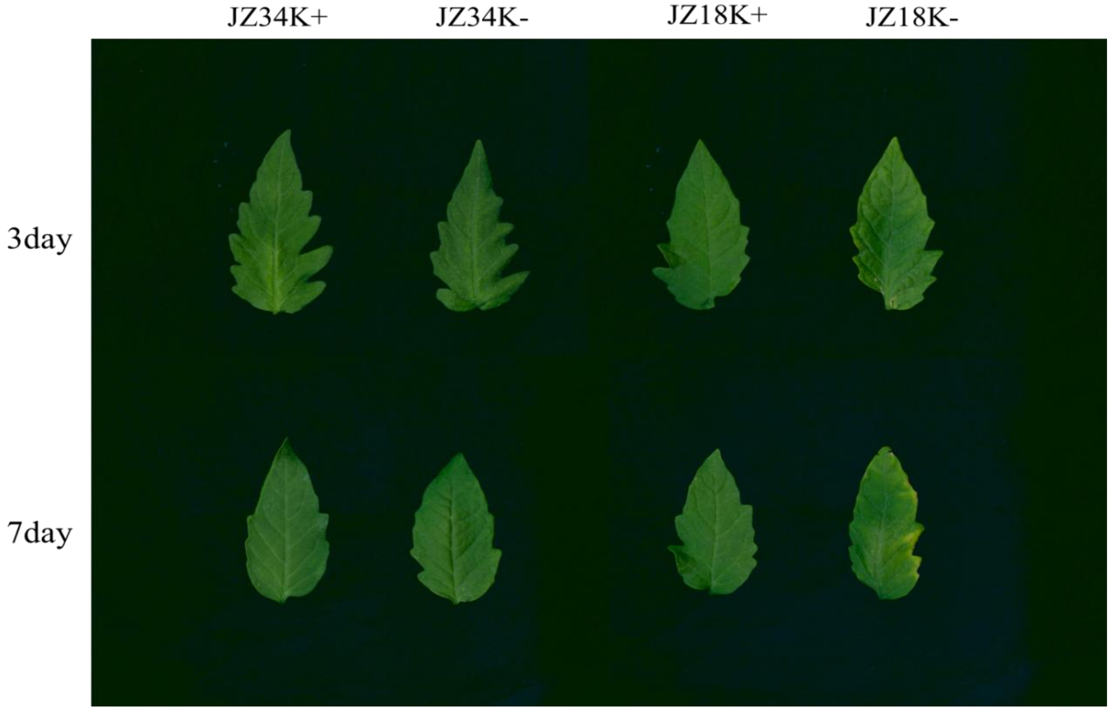

K+ represents normal K^+; K- represents K^+ deficiency
