# Supplementary material for: Comparative Transcriptome Profiling of Two Tomato Genotypes in Response to Potassium-Deficiency Stress
Source: Int J Mol Sci. 2018 Aug 14;19(8):2402. doi: 10.3390/ijms19082402 (PMC6121555; doi:10.3390/ijms19082402)

Figure S2: Hierarchical clustering analysis of the oppositely regulated DEGs under K^+-deficiency stress in the two varieties.

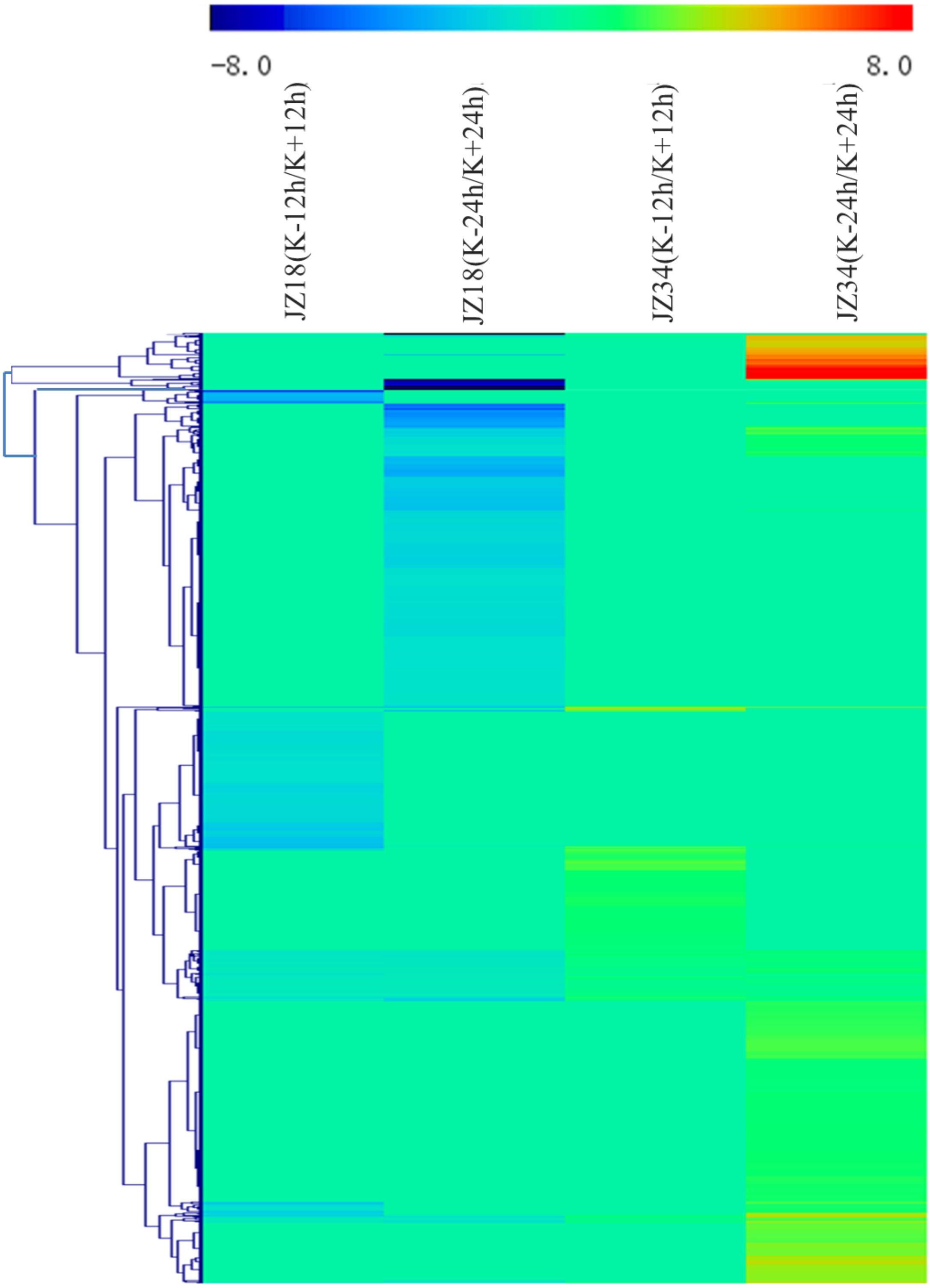

Supplement: Supplementary file 1 [file ijms-19-02402-s001.zip › ijms-323017 supplementary update/Figure S2.pdf]
