# Supplementary material for: Comparative Transcriptome Profiling of Two Tomato Genotypes in Response to Potassium-Deficiency Stress
Source: Int J Mol Sci. 2018 Aug 14;19(8):2402. doi: 10.3390/ijms19082402 (PMC6121555; doi:10.3390/ijms19082402)
Supplement: Supplementary file 1 [file ijms-19-02402-s001.zip › ijms-323017 supplementary update/Table S1.pdf]

Table S1: Gene function annotations of 20 DEGs

Transcript ID	Description
Solyc01g010480.2.1	Potassium channel
Solyc03g097930.2.1	potassium channel SKOR-like
Solyc07g014680.2.1	Potassium voltage-gated channel
Solyc11g011500.1.1	KUP system potassium uptake protein
Solyc12g009540.1.1	Potassium transporter
Solyc03g005520.1.1	Ethylene responsive transcription factor 1a
Solyc03g005500.1.1	Ethylene-responsive transcription factor 14
Solyc02g094270.1.1	WRKY transcription factor
Solyc03g082430.1.1	Growth-regulating factor 4
Solyc04g074030.2.1	LRR receptor-like protein kinase
Solyc12g009780.1.1	LRR receptor-like protein kinase
Solyc01g006390.2.1	Cysteine-rich receptor-like protein kinase
Solyc01g068410.1.1	Auxin Efflux Carrier
Solyc10g017990.1.1	Cytokinin dehydrogenase 1
Solyc07g052370.2.1	Cytochrome P450
Solyc06g066230.2.1	Cytochrome P450
Solyc07g056430.2.1	Glutathione S-transferase
Solyc07g056510.2.1	Glutathione S-transferase-like protein
Solyc03g097560.2.1	bidirectional sugar transporter SWEET14
Solyc08g066100.2.1	ATP-dependent 6-phosphofructokinase
