# Supplementary material for: Comparative Transcriptome Profiling of Two Tomato Genotypes in Response to Potassium-Deficiency Stress
Source: Int J Mol Sci. 2018 Aug 14;19(8):2402. doi: 10.3390/ijms19082402 (PMC6121555; doi:10.3390/ijms19082402)
Supplement: Supplementary file 1 [file ijms-19-02402-s001.zip › ijms-323017 supplementary update/Table S2.pdf]

Table S2: DEGs related to oxidative proteins in response to K^+-deficiency stress.

Group	Gene ID	log2(Fold change)				seq description
		JZ18		JZ34		
		12h	24h	12h	24h	
peroxidase	Solyc01g015080.2.1	1.127694108				Peroxidase
	Solyc02g090450.2.1	1.073084029				Peroxidase
	Solyc06g050440.2.1	-1.094105721				Peroxidase
	Solyc10g076190.1.1	-1.18674262				Peroxidase 1
	Solyc10g076210.1.1	-1.25698262				Peroxidase 1
	Solyc02g084780.2.1	-1.323509333				Peroxidase
	Solyc02g084800.2.1	-1.328929699				Peroxidase
	Solyc02g084790.2.1	-1.368297493				Peroxidase
	Solyc07g056480.2.1	-1.63917944				Peroxidase
	Solyc04g054500.2.1	-1.639433702				Peroxidase
	Solyc01g101050.2.1		1.471219355			Peroxidase 1
	Solyc03g025380.2.1		1.270569789			Peroxidase
	Solyc12g005790.1.1		1.199173712			Peroxidase 27
	Solyc11g018800.1.1		1.192939395			Peroxidase 2
	Solyc11g012690.1.1		1.141147717			Peroxidase
	Solyc11g010120.1.1		-1.068593205			Peroxidase 17
	Solyc01g105770.2.1		-1.089735273		1.111556848	Peroxidase
	Solyc04g081860.2.1		-1.288512683			Peroxidase
	Solyc11g069070.1.1		-1.397682167			Peroxidase
	Solyc07g049240.2.1		-1.422905743			Peroxidase
	Solyc05g046030.2.1		-1.630585118		1.313263234	Peroxidase
	Solyc01g067850.2.1			2.934371332		Peroxidase
	Solyc05g054250.1.1			1.152239194		Peroxidase
	Solyc01g067860.2.1			1.101595196	1.057419063	Peroxidase 24
	Solyc12g005790.1.1			-1.004196657		Peroxidase
	Solyc04g015030.2.1			-1.648348065		Peroxidase
	Solyc12g096530.1.1			-1.791111843		Peroxidase
	Solyc05g010330.2.1				4.392317423	Peroxidase
	Solyc10g084240.1.1				1.438213822	Peroxidase
	Solyc01g067850.2.1				2.584962501	Peroxidase
	Solyc04g071890.2.1				2.06523595	Peroxidase 4
	Solyc04g081860.2.1				1.472241675	Peroxidase
	Solyc05g046020.2.1				1.348931522	Peroxidase
	Solyc03g006700.2.1				1.346069494	Peroxidase
	Solyc08g013930.2.1				1.342017247	Peroxidase
	Solyc02g092580.2.1				1.30462536	Peroxidase
	Solyc05g050880.2.1				1.252496046	Peroxidase
	Solyc05g046010.2.1				1.140662496	Peroxidase
	Solyc07g017880.2.1				1.133880935	Peroxidase

	Solyc12g005370.1.1				1.133423897	Peroxidase 27
	Solyc03g044100.2.1				1.068666021	Peroxidase 5
	Solyc10g076210.1.1				-1.059655666	Peroxidase 1
Cytochrome P450	Solyc07g052370.2.1	1.217845632			6.135281758	Cytochrome P450
	Solyc03g114940.2.1	1.183381282	-2.633525466			Cytochrome P450
	Solyc04g078290.2.1	-1.232265837			1.418532337	Cytochrome P450
	Solyc11g030730.1.1	-1.42752013	1.364363361			Cytochrome P450
	Solyc04g078340.2.1	-1.504512668				Cytochrome P450
	Solyc10g007890.2.1	-1.637515507				Cytochrome P450
	Solyc03g122350.2.1	-1.647956904				Cytochrome P450
	Solyc03g111880.2.1	-1.793477615				Cytochrome P450
	Solyc07g041500.2.1	-2.189421177				Cytochrome P450
	Solyc07g006140.2.1	-2.643513059				Cytochrome P450
	Solyc06g074420.1.1		2.369534027		1.676844602	Cytochrome P450
	Solyc04g079730.1.1		2.149299481	1.329920886		Cytochrome P450
	Solyc03g111300.1.1		2.115477217			Cytochrome P450
	Solyc02g094110.1.1		1.40730185	1.915839552		Cytochrome P450
	Solyc02g080330.2.1		-1.209141398	-2.461522987	-1.545717123	Cytochrome P450
	Solyc05g015350.2.1		-1.298105219			Cytochrome P450
	Solyc03g122360.2.1		-1.392317423			Cytochrome P450
	Solyc12g088970.1.1			2.274106664		Cytochrome P450
	Solyc07g043460.2.1			1.285376368		Cytochrome P450
	Solyc01g080900.2.1			1.091350462		Cytochrome P450
	Solyc02g065190.2.1			1.029570322		Cytochrome P450
	Solyc10g017510.2.1			1.063999516		Cytochrome P450
	Solyc01g008650.2.1			-1.142604395		Cytochrome P450
	Solyc06g076160.2.1			-1.154658245	4.943979914	Cytochrome P450
	Solyc10g083400.1.1			-1.291956015		Cytochrome P450
	Solyc08g081220.1.1			-1.401721298		Cytochrome P450
	Solyc04g083140.1.1			-1.477321778		Cytochrome P450
	Solyc09g059240.2.1			-1.498805857		Cytochrome P450
	Solyc03g112030.1.1			-1.543142325	2.446952679	Cytochrome P450
	Solyc10g078230.1.1			-1.987509056		Cytochrome P450
	Solyc10g080870.2.1			-2.095507331	-2.222392421	Cytochrome P450
	Solyc10g081550.1.1			-2.224966365		Cytochrome P450
	Solyc04g071780.2.1			-2.373458396	1.887525271	Cytochrome P450
	Solyc06g073570.2.1			-2.620151929		Cytochrome P450
	Solyc06g066230.2.1				6.185866545	Cytochrome P450
	Solyc05g047530.2.1				4.02128092	Cytochrome P450
	Solyc04g083140.1.1				3.837484588	Cytochrome P450
	Solyc01g109140.2.1				1.73875564	Cytochrome P450
	Solyc02g090350.2.1				1.73104076	Cytochrome P450
	Solyc05g021390.2.1				1.607122567	Cytochrome P450

	Solyc06g082730.2.1				1.424922088	Cytochrome P450
	Solyc03g112040.1.1				1.374395515	Cytochrome P450
	Solyc06g076800.2.1				1.261086134	Cytochrome P450
	Solyc04g071800.2.1				1.179835021	Cytochrome P450
Glutathione S-transferase	Solyc07g056440.2.1	-1.066005681				Glutathione S-transferase-like protein
	Solyc06g069040.2.1	-1.309328058				Glutathione S-transferase
	Solyc07g056500.2.1	-1.358781819				Glutathione transferase
	Solyc12g011300.1.1	-1.482931534				Glutathione transferase
	Solyc07g056420.2.1	-1.591337951				Glutathione S-transferase-like protein
	Solyc10g084400.1.1	-1.633084981				Glutathione S-transferase
	Solyc07g056480.2.1	-1.63917944				Glutathione S-transferase-like protein
	Solyc07g056470.2.1	-1.782755945	-1.018378529			Glutathione S-transferase-like protein
	Solyc01g086680.2.1	-1.795800695				Glutathione S-transferase
	Solyc09g011630.2.1	-1.981746452				Glutathione S-transferase-like protein
	Solyc09g011590.2.1	-2.291763291			-1.479196505	Glutathione S-transferase-like protein
	Solyc09g011520.2.1	-2.450679698				Glutathione S-transferase-like protein
	Solyc06g009040.2.1	-2.605426603				Glutathione S-transferase
	Solyc09g074850.2.1	-2.776778074			-1.503701886	Glutathione S-transferase
	Solyc09g011620.1.1	-4.591089973				Glutathione S-transferase-like protein
	Solyc06g075520.2.1		2.111446536			Glutathione S-transferase
	Solyc02g081340.2.1		-1.346450414			Glutathione S-transferase
	Solyc07g056460.2.1		-1.556049809			Glutathione S-transferase-like protein
	Solyc09g011540.2.1		-2.092649288			Glutathione S-transferase-like protein
	Solyc07g056510.2.1				7.64385619	Glutathione S-transferase
	Solyc07g056430.2.1				7.087462841	Glutathione S-transferase-like protein
	Solyc09g011550.2.1				3.03678667	Glutathione S-transferase-like protein
	Solyc03g116120.1.1				-3.010569242	Glutathione S-transferase 12
