# Supplementary material for: Comparative Transcriptome Profiling of Two Tomato Genotypes in Response to Potassium-Deficiency Stress
Source: Int J Mol Sci. 2018 Aug 14;19(8):2402. doi: 10.3390/ijms19082402 (PMC6121555; doi:10.3390/ijms19082402)
Supplement: Supplementary file 1 [file ijms-19-02402-s001.zip › ijms-323017 supplementary update/Table S5.pdf]

Table S5: Primers used for qRT-PCR.

	Gene Name	Primers(5`-3`)
A	Solyc01g010480	F:AGAAGGACGGGAGAGTGAT<br>R:TGAACCTGATGCCAGAGA
B	Solyc03g097930	F:CGTCCCCTGCTTCTACTACT<br>R:AGAATCGGTTGAAGCTGCTG
C	Solyc07g014680	F:CCTACCGTCTTTTCGTCCTC<br>R:GCTTCCCCACCAAGAAACAT
D	Solyc11g011500	F:TCTGGAGGAGGTTGGTATTG<br>R:GGCAAATGACTGCTTATCG
E	Solyc12g009540	F:TGGAGAGTTGGAGGAAGAGAT<br>R:TACCACTGCCTTGGAGACTG
F	Solyc03g005520	F:CTATGACCTTCTCGCACAAG<br>R:TCAGCACTATCAAATGTCCC
G	Solyc03g005500	F:TCGTGTTTGGCTAGGGACAT<br>R:TATGGCCAAATGTCCCCTCA
H	Solyc02g094270	F: AGGGTTTCTTGGGATTCG<br>R: TCCTTATTTCGTGTTCGGTC
I	Solyc03g082430	F:TTCTTCCTCCACCTCCTTC<br>R:GTAGTCATTCCTCCTTGGGA
J	Solyc04g074000	F:CAACTTCTCTGGCACTATCCC<br>R:GTTTCCCTATTTCTGCTGGA
K	Solyc04g074030	F:CAATGGCGAAGGGACATA<br>R:AGAACCAGCGAAAGCACA
L	Solyc12g009780	F:TGTCAACATCTCTTCTCCGA<br>R:TCAATGAACTCAAGGTGCC
M	Solyc01g006390	F:GGTCATGGCAAGAGAAGTCG<br>R:AACTTGTTGGTGAGGGTGGA
N	Solyc10g017990	F:AGGGTTTCTTGGGATTCG

		R:TCCTTATTTCGTGTTCGGTC
O	Solyc07g052370	F:GGTCAAGTCATTCAGCCCT
		R:GCGGCATTCATAAACCATAG
P	Solyc06g066230	F:TGGAGGTGAAGATTTGGTTG
		R:TCAGTCCCAGCGGTAAACA
Q	Solyc07g056430	F:GCACTTGGAGAGAAGCCTT
		R:CTTTGTATGCTGCGGATG
R	Solyc07g056510	F:CCTTTGGTATGAGGGTGAGA
		R:GGATTTGCTTATGGATAGGG
S	Solyc03g097560	F:GGGTTCCAATCAGTTCCTT
		R:GTTTCATCGTGTCCATCTTG
T	Solyc08g066100	F:TGAAGGTGGTGAATGGAGA
		R:TATGCTGGATTAGACCGCA
	Actin	F: TGTCCCTATTTACGAGGGTTATGC
		R: AGTTAAATCACGACCAGCAAGAT
